# Social relations and healthcare utilisation among middle-aged and older people: study protocol for an implementation and register-based study in Denmark

**DOI:** 10.1186/s12913-017-2650-0

**Published:** 2017-11-15

**Authors:** Anne Sophie Bech Mikkelsen, Rikke Lund, Maria Kristiansen

**Affiliations:** 1 Unit for Health Services Research and Unit for Social Medicine, Department of Public Health, Center for Healthy Aging, University of Copenhagen, Copenhagen, Denmark; 2 Unit for Social Medicine, Department of Public Health, Center for Healthy Aging, University of Copenhagen, Copenhagen, Denmark

**Keywords:** Social relations, Healthcare utilisation, Loneliness, Aging, Implementation, Complex interventions

## Abstract

**Background:**

While previous research establishes an association between social relations, health and use of healthcare services among older people, how to implement this knowledge in real-life settings has received much less attention. This study will explore the relationship between social relations, health and use of healthcare services in a Danish mid-life population sample. In addition, the study will explore individual and contextual factors affecting the implementation of a group-based life story intervention aimed at establishing and strengthening social relations among older people at nursing homes in Denmark.

**Methods/design:**

A combined quantitative register-based approach and a qualitative implementation approach will be applied in this study. First, we will quantitatively analyse the relationship between social relations, health status and use of healthcare services among middle-aged people in Denmark by linking survey data on social relations, loneliness, self-perceived health and disease status from the Copenhagen Aging and Midlife Biobank (CAMB) (*n* = 7191) with national registries through the Public Health Database on use of healthcare services and demographic and socioeconomic factors. Second, we will qualitatively analyse individual and contextual factors affecting the implementation process of the group-based life story intervention based on semi-structured interviews (*n* = 16), observations and field notes with and among intervention stakeholders, i.e., participants and group leaders facilitating the intervention.

**Discussion:**

The results of this study are expected to improve knowledge about mechanisms through which social relations are associated with health status and use of healthcare services and to inform the implementation of future interventions targeting social relations among older people at nursing homes.

**Trial registration:**

The study has been registered and approved by the Danish Data Protection Agency. Seperate approvals have been attained for the qualitative data (Approval No. SUND-2016-08), and for the quantitative data in the CAMB database which has also received approval from the local ethical committee (approval No.H-A-2008-126 and No. 2013–41-1814, respectively).

## Background

Previous research establishes an association between social relations, health status and use of healthcare services among older people. Different hypotheses of mechanisms through which both functional and structural aspects of social relations act have been studied both quantitatively and qualitatively. In general, strong social relations have been found to have a positive effect on health measures such as morbidity, mortality, well-being, survival after illness and recovery, as well as on the use of healthcare services. Weak or strained social relations, on the other hand, have been found to have a negative effect on these measures [1–4]. Additionally, there has been an increasing focus in recent years on loneliness among older people and on the negative effect that loneliness has on health. The causes of loneliness among older adults are complex and multifactorial; loneliness has been found to interact with terms such as social relations, feeling unwanted alone, and social isolation in shaping health and use of healthcare services [5–8].

There is an interest in moving this knowledge from research to practice in different settings. The rationale for implementing interventions that target social relations and doing this specifically in a group-based manner seems to be supported by findings from recent studies that demonstrate that group-based social interventions targeting loneliness, social isolation and social support can have a positive impact on subjective health, on the use and cost of healthcare services and on mortality [6, 9, 10]. Interventions addressing the social aspects of individuals’ lives can often be considered complex in the sense that what such interventions wish to address and change might rely heavily on the embedded and habitual behaviours of both the participants/end users of the intervention and the individuals engaged in implementation of the intervention [11, 12]. The complex intervention framework acknowledges issues of complexity, context and the interplay of intervention components, which then become key factors in analyses of implementation in order to generate knowledge about what works for whom and under what circumstances [13–15]. Based on this, we find it relevant to further explore the relationship between social relations, health status and use of healthcare services among middle-aged and older people, and to explore the implementation of interventions targeting social relations among older people living at nursing homes.

Our study will first explore the relationship between social relations, health status and use of healthcare services in a large middle-aged population sample using a social epidemiological approach. Second, we will explore individual and contextual factors affecting the implementation process of a group-based life story intervention aimed at establishing and strengthening social relations among older people at nursing homes. This will be accomplished by adopting a qualitative longitudinal approach based on semi-structured interviews and observations conducted with intervention stakeholders (participants and group-leaders facilitating the intervention) throughout the intervention period. Based on analyses and findings from these two sub-studies, we will then reflect upon the potential of interventions to support social relations in more vulnerable groups of older adults in nursing home settings, and more broadly how interventions to enhance social relations may affect health status and use of healthcare services in the different phases of aging.

The following research questions are designed to explore the relationship between social relations, health status and use of healthcare services:

- What is the relationship between social relations, health status and use of healthcare services?
- How is the relationship between social relations and use of healthcare services modified by loneliness?

The following research questions of the study are designed to explore individual and contextual factors affecting the implementation of the group-based life story intervention in order to establish and strengthen social relations among older people:

c. What are participants’ and group-leaders’ experiences with the implementation?
d. How are the implementation processes affected by individual factors and contextual factors?

## Methods/design

First, we will quantitatively analyse the relationship between social relations, health status and use of healthcare services among middle-aged people in Denmark. Second, we will qualitatively analyse individual and contextual factors affecting the implementation process of the group-based life story intervention. Accordingly, the study is divided into two sub-studies. Table 1 provides an overview of aim, research questions, methodologies, data sources and analysis of each of the sub-studies. Each of the sub-studies will be described in the following sections.

**Table 1:** Aims, methodologies, data sources and analyses applied

Aim and sub-study	Research question	Methodology	Data source	Analysis
Explore the relationship between social relations, health status and use of healthcare services among middle-aged people	What is the relationship between social relations, health status and use of healthcare services?	Social epidemiological approach based on analyses in dataset consisting of survey and register data	Copenhagen Aging and Midlife Biobank (CAMB) and national register data from the Public Health Database (*n* = 7191)	Descriptive and multivariate correlation analyses
	How is the relationship between social relations, health status and use of healthcare services modified by loneliness?	Social epidemiological approach based on analyses in dataset consisting of survey and register data	Copenhagen Aging and Midlife Biobank (CAMB) and national register data from the Public Health Database (n = 7191)	Descriptive and multivariate correlation analyses
Explore individual and contextual factors affecting implementation of the group-based life story intervention aimed at establishing and strengthening social relations among older people at nursing homes	What are participants’ and group-leaders’ experiences with the implementation?	Multi-perspective longitudinal approach conducting qualitative semi-structured interviews and participant observations	Intervention participants and group-leaders (*n* = 16)	Qualitative content analysis
	How are the implementation processes affected by individual factors and contextual factors?	Multi-perspective longitudinal approach conducting qualitative semi-structured interviews and participant observations	Intervention participants and group-leaders (n = 16)	Qualitative content analysis

### Sub-study 1: Quantitative data collection and analysis

#### Theoretical framework

The concept of social relations and their implications for healthcare utilisation is the overarching theme of this study, which makes it relevant to outline clearly how we define and utilise the concept. The concept of social relations has been defined as comprising both structural and functional aspects. The structure of an individual’s social life refers to the size of an individual’s social network, and its diversity, density, reciprocity and frequency of contacts, while the function of social relations refers to emotional and instrumental support, social anchorage and relational strain. Relational strain may be caused by experienced conflicts and excessive demands from the individual’s social relations, which have been found to have a negative effect on health. As needs and resources change over the course of life, the structure and function of one’s social relations and the experience of relational strain may change: they are not static phenomena, but rather may be specific to different phases of life [4, 16]. Social relations may shape healthcare use by providing transport to healthcare facilities, assisting in navigating complex healthcare systems, seeking information, providing emotional support throughout a treatment period and supporting patients in disease management through, for example, the taking of medications or sustaining a certain health-promoting behaviour. If social relations are not accessible and/or are not providing sufficient support, or if the individual is not anchored in a supportive network or group, accessing healthcare services may be hindered because of a lack of sufficient emotional and structural support and the resources required to seek healthcare services. Moreover, social relations have been found to interact in a complex way with related terms such as loneliness, feelings of being unwanted alone and social isolation that affect both health and use of healthcare services [4, 16]. Building on this existing literature our main hypothesis is, that strong and/or not straining social relations improve health and reduce healthcare utilisation in a mid-life Danish population.

#### Methods and material – The Copenhagen aging and midlife biobank (CAMB) and the public health database (PHD)

The Copenhagen Aging and Midlife Biobank (CAMB) has been established to contribute new knowledge on the life-course determinants of health and early aging in mid- and late life, and is designed with particular emphasis on the earlier stages of the aging process. CAMB includes participants from three earlier established cohort studies with data on the first half of life: The Metropolit 1953 Danish Male Birth Cohort (MP), The Copenhagen Perinatal Cohort (CPC) (born 1959–61), and the Danish Longitudinal Study on Work, Unemployment and Health (DALWUH) (born 1949–1959). In total, 17,937 cohort members (aged 49–61) were invited to participate in the study (MP: 7750, CPC: 5282, and DALWUH: 4906); 7191 (40%) (4954 men and 2234 women) completed a postal questionnaire covering psychosocial, behavioural, health-related and social variables and 5576 (31%) (3819 men and 1754 women) in addition participated in a clinical examination as well as physical and cognitive tests. Baseline information about respondents and non-respondents was retrieved from Statistics Denmark [17].

Through the CAMB survey data collected in 2009–2011 and register data we will explore the association between social relations, health status and use of healthcare services in a 2 year follow-up period. We will explore whether and how loneliness and gender modifies this association. Data on social relations, loneliness, self-perceived health and disease status from the Copenhagen Aging and Midlife Biobank (CAMB), will, through the Civil Registration System (CPR), be linked to register data on the use of healthcare services and demographic and socioeconomic factors from the Public Health Database situated at the Department of Public Health, University of Copenhagen [18]. The measures from CAMB on self-perceived health and disease status (self-reported) will be used as proxies for health status. Data on use of healthcare services retrieved from registries will be measured in our study in terms of type and frequency of contact with a general practitioner, and the number and duration of hospital contacts and admissions.

An epidemiological approach will guide the analytical strategy in the statistical analyses, in which social relations will be considered to be the main exposure variable affecting the main outcome variable, that is, use of healthcare services. Self-perceived health, disease status, loneliness and gender will be analysed as effect-modifiers by conducting sub-group analyses. Socioeconomic and demographic factors will be analysed as possible confounding variables. Additional data on variables such as living conditions, vitality, stress, fatigue, sleeping disturbances, level of physical functioning, social capital in the local community, social class, social position, sick days, sexual life, severe life events (in childhood, adulthood or working life), smoking, alcohol consumption and level of physical activity will be available through the survey data and will be included in a model search to assess whether they might be relevant to include as possible confounding variables [19]. Initial analyses will be descriptive and univariate, followed by multivariate analyses using a Poisson regression model to handle the outcome count variables (number of contacs to general practitioner and to hospital). Different length of follow-up due to for example death or emmigration before end of the 2 year follow-up period will be handled by including an offset variable in the Poisson regression model by which information on participants leaving the study are not excluded from the analyses. SAS statistics will be used to process the data via remote access to Statistics Denmark’s server, and all hypotheses will be tested at a 5% significance level (*p* < 0.05).

We use the power analysis program G*Power developed and described by Faul et al. [20] to perform power calculations. With a total sample size of 7191 cases, our calculations suggest that t-tests and F-tests with up to 15 covariates will be able to detect effect sizes as low as parameter estimates of 0.001 and increases in the coefficient of determination of 0.003 at a significance level of 0.05 with a power of 0.8. Effect sizes of that magnitude fall well below the threshold of theoretically interesting relationships. In other words, lack of power is not likely to be a problem for the statistical analyses.

### Sub-study 2: Qualitative data collection and analysis

#### Theoretical framework

In order to analyse factors affecting the implementation process of the group-based life story intervention, we will apply the Normalization Process Theory (NPT) developed by Carl May [11, 12, 21]. Interventions in healthcare and social settings engage multiple stakeholders, rely on often embedded and habitual behaviours, and are often implemented in varying contexts and enrol participants with varying characteristics [11, 12]. Hence, we find it feasible to apply a theory such as the NPT, which deals specifically with complex interventions. The core components of the NPT are: the *capabilities* inherent in the intervention (qualities of workability and integration in practice), the *contribution* to embedding or enacting the complex intervention into daily practice (coherence/sense-making, cognitive participation/engagement, collective action and reflexive monitoring), the *potential* to translate capacity into action (readiness of practitioners to make contributions to the complex intervention) and the *capacity* to cooperate and coordinate action amongst practitioners (rules and roles that govern behaviour around the complex intervention) [11, 12, 21]. According to the NPT, the implementation of a complex intervention such as the group-based intervention to be addressed in this study depends on the capabilities of the intervention and the contributions of the practitioners implementing the intervention, i.e., the healthcare professionals facilitating the group meetings. Furthermore, according to the NPT, practitioners’ contributions to the implementation might be affected by their capacity and potential to engage with the implementation. We will also consider participants’ actions and experiences of the implementation throughout all the components of the NPT. How we will address these four components is elaborated in the methodology section.

#### The group-based life story intervention

The intervention in this study will serve as a concrete example of group-based interventions that target social relations, or similar aspects of the individuals’ social lives. The group-based life story intervention to be addressed in this study was developed by the private non-profit EGV Foundation [22], which facilitates the social inclusion of older people. After pilot testing the intervention among community-dwelling older adults and receiving a positive evaluation the EGV Foundation is now partnering with Copenhagen Municipality to implement it in several local nursing homes in Copenhagen. This setting implies a shift in target group as older adults in nursing homes are in general more frail than community-dwelling older adults. There is therefore a need for exploring transferability of the intervention to this new setting and population.

The intervention’s theoretical foundation rests in narrative therapy, which sees a potential for change in the process of sharing significant life stories. The core intervention activity involves facilitating and supervising group meetings organised around participants sharing their life stories. The group leaders facilitating and supervising the group meetings are healthcare professionals employed at the nursing homes who have undergone a three-day training course organised by the EGV Foundation on how to recruit participants, facilitate the group meetings, supervise and follow-up, if needed. The joint implementation by the EGV Foundation and Copenhagen Municipality began in spring 2016, and will continue throughout 2017, with each of the intervention groups meeting for eight to ten weeks, plus a follow-up period in which the focus will be on supporting volunteer- and participant-run continuation of the activities. The intervention focuses on social relations not merely as present or established during the intervention activities, but rather as those that will last and be sustained beyond the intervention period. The rationale of the intervention is that sharing life stories during supervised and facilitated group meetings can establish and strengthen social relations and prevent or reduce feelings of loneliness. This is based on an assumption that bringing older people together in smaller groups creates a space where they, by sharing their life stories, can maintain links to their personal biography. As the EGV Foundation states in their description of the intervention: ‘Through stories and social context, the participants connect, they become new witnesses to each other’s life stories and they establish a new outlet for social relations’ [22].

#### Methods and material

A multi-perspective longitudinal approach is used in the qualitative sub-study. Participants and group leaders facilitating the intervention group meetings are interviewed prior to and after the intervention period. Since we are interested in exploring the implementation process of the intervention, the selection of implementation settings (nursing homes) and informants at the seleced settings (both participants and group leaders) are depending on gaining access through different gatekeepers. The implementation partners have themselves decided at which nursing homes to implement the intervention and together with management level at the nursing homes they have decided on who among the staff should be invited for the three-day training course. We have made initial field observations at the three-day training course at which we presented ourself and our reason for being there. The EGV Foundation carried out the training course and served as a gatekeeper for gaining access to group leaders wishing to participate in the study. Three group leaders agreed to participate out of which two of them work at the same nursing home and are to facilitate a group together. This way two specific nursing homes have been selected for the study. As part of their group leader training, the healthcare professionals were themselves responsible for inviting and selecting 4–5 participants for their individual intervention groups. All participants were given a information folder about this study including contact information on the researcher conducting the interviews and observation. All participants were asked by the group leaders to be part of the study and from each of the groups two participants agreed to participate in interviews. This way the group leaders serve as gatekeepers for selecting intervention participants to be included in the study. However, observations were made among all participants in the group sessions upon informed consent. Interviews with participants are conducted in their private domain in their nursing homes, and interviews with group leaders take place in a meeting room at the nursing homes. All interviews will be carried out in a semi-structured fashion using pre-developed flexible interview guides, and will be recorded, transcribed and subsequently organised, coded and analysed in Nvivo [23].

Coding and analyses will be done based on the predefined themes as described in the applied Normalization Process Theory (NPT). The *capabilities* of the intervention itself will be addressed with a focus on how the healthcare professionals and participants engage in implementing the intervention, how they enact the intervention, how the healthcare professionals have been trained to do so and with a focus on confidence in its use and the resources that are available to execute the intervention. These elements will be addressed through observation notes from the three-day training course and pre- and post intervention semi-structured interviews with the group leaders and participants. We expect to uncover elements of the group leaders’ engagement in the intervention and how these might have affected the implementation of the intervention – in either an inhibiting or facilitating manner [11, 12, 21].

The second component of the NPT relating to determinants *contributing* to embedding the intervention into practice will be addressed by focusing on the group leaders’ and participants’ contributions to enacting the intervention. According to the NPT, group leaders’ contributions can be identified as what they do when interacting with colleagues vis-à-vis the context in which the intervention is implemented. In line with this we will also focus on how participants’ interactions with other participants and with group leaders might affect the implementation. We will seek to uncover how their ways of interacting with colleagues at the nursing homes, i.e., other healthcare professionals and managers, and their daily reflections about the effects of the intervention might affect its implementation. This will be accomplished through observation notes from the facilitated group meetings and from informal meetings at the implementation sites, together with pre- and post- semi-structured interviews with the group leaders and participants [11, 12, 21].

The third and fourth components of the NPT address the *capacity* of group leaders to cooperate and coordinate action relating to the embedding of the intervention and their *potential* to enact and engage with the intervention. Capacity relates to structural resources such as setting characteristics, e.g., the culture, value and norms of the setting in which the intervention is implemented and enacted – in this case, nursing homes. *Potential* relates to cognitive resources, which is understood as individual psychological resources that constitute individuals’ readiness to participate in implementation. Here, we will also see actions and experiences of the intervention participants as part of available cognitive resources affecting the implementation of the intervention. The structural resources are part of what constitutes the conditions in which the healthcare professionals work and where they seek to embed the intervention. The structural and cognitive resources of the healthcare professionals and the participants will be addressed by pre- and post- intervention interviews, and informal talks and meetings with the group leaders engaged in implementing the intervention and with participants in the intervention [11, 12, 21].

## Discussion

Based on a considerable amount of data, we expect this study to generate knowledge about the relationship between social relations, health status and use of healthcare services. We also expect that the role of loneliness on individuals’ use of healthcare services will be explored and clarified. Additionally, the study will contribute valuable knowledge about what individual and contextual influences affect the implementation of group-based interventions targeting social relations among vulnarable older people at nursing homes. Local differences at both individual and contextual levels make it relevant to focus on the tension between intervention fidelity and adaptation to local contexts when assessing the success of an implementation process. Dissemination of findings will be conducted through scientific publications as well as through presentations for key intervention stakeholders at the national and international levels.

## Abbreviations

CAMB: Copenhagen Aging and Midlife Biobank
EGV: Foundation for Social Inclusion of Older Adults
NPT: Normalization Process Theory
PHD: Public Health Database
SAS: Statistical Analysis System

## References

[1] Wolinsky FD, Johnson RJ (1991). The use of health services by older adults. J Gerontol.

[2] Cohen S (2004). Social relationships and health. Am Psychol.

[3] Kuiper JS, Zuidersma M, Oude Voshaar RC (2015). Social relationships and risk of dementia: a systematic review and meta-analysis of longitudinal cohort studies. Ageing Res Rev.

[4] Lund R, Nielsen LS, Henriksen PW (2014). Content validity and reliability of the Copenhagen social relations questionnaire. J Aging Health.

[5] Hawkley LC, Cacioppo JT (2010). Loneliness matters: a theoretical and empirical review of consequences and mechanisms. Ann Behav Med.

[6] Pitkala KH, Routasalo P, Kautiainen H (2009). Effects of psychosocial group rehabilitation on health, use of health care services, and mortality of older persons suffering from loneliness: a randomized, controlled trial. J Gerontol A Biol Sci Med Sci.

[7] Savikko N. Loneliness of Older People and Elements of an Intervention for its Alleviation. Published Online First: 15 August 2008. http://www.doria.fi/handle/10024/38910. Accessed 11 Sept 2015.

[8] Schirmer W, Michailakis D. Loneliness among older people as a social problem: the perspectives of medicine, religion and economy. Ageing Soc. 2015;FirstView:1–21. doi:10.1017/S0144686X15000999

[9] Hogan BE, Linden W, Najarian B (2002). Social support interventions: do they work?. Clin Psychol Rev.

[10] Dickens AP, Richards SH, Greaves CJ (2011). Interventions targeting social isolation in older people: a systematic review. BMC Public Health.

[11] Richards DA. Complex Interventions in Health. An overview of research methods. Taylor Francis EBooks. http://www.tandfebooks.com.ep.fjernadgang.kb.dk/doi/book/10.4324/9780203794982. Accessed 24 Nov 2016.

[12] Nilsen P (2015). Making sense of implementation theories, models and frameworks. Implement Sci.

[13] Bonell C, Fletcher A, Morton M (2012). Realist randomised controlled trials: a new approach to evaluating complex public health interventions. Soc Sci Med.

[14] Pinnock H, Epiphaniou E, Sheikh A (2015). Developing standards for reporting implementation studies of complex interventions (StaRI): a systematic review and e-Delphi. Implement Sci.

[15] Marchal B, Westhorp G, Wong G (2013). Realist RCTs of complex interventions – an oxymoron. Soc Sci Med.

[16] Due P, Holstein B, Lund R (1999). Social relations: network, support and relational strain. Soc Sci Med.

[17] Lund R, Mortensen EL, Christensen U, et al. Cohort Profile: The Copenhagen Aging and Midlife Biobank (CAMB). Int J Epidemiol. 2015;:dyv149. doi:10.1093/ije/dyv149 10.1093/ije/dyv149 26210613

[18] Lynge E, Sandegaard JL, Rebolj M (2011). The Danish National Patient Register. Scand J Public Health.

[19] Rothman KJ. Epidemiology, an introduction. 2 ed. New York: Oxford University Press; 2012.

[20] Faul F, Erdfelder E, Lang A-G (2007). G*power 3: a flexible statistical power analysis program for the social, behavioral, and biomedical sciences. Behav Res Methods.

[21] May C, Finch T (2009). Implementing, embedding, and integrating practices: an outline of normalization process theory. Sociology.

[22] English | Ensomme Gamles Værn. http://www.egv.dk/en/. Accessed 29 Nov 2016.

[23] Madden R. Being ethnographic, a guide to the theory and practice of ethnography. London: SAGE Publications Ltd.; 2010.

[24] WMA Declaration of Helsinki - Ethical Principles for Medical Research Involving Human Subjects. 2013.https://www.wma.net/policies-post/wma-declaration-of-helsinki-ethical-principles-for-medical-research-involving-human-subjects/. Accessed 18 Mar 2016.

